# Gastric Dyspepsia

**Published:** 1882-07

**Authors:** William Henry

**Affiliations:** Harmon Lee Co., Ill.


### ARTICLE VI.

GASTRIC DYSPEPSIA. By WILLIAM HENRY, M.D., Harmon Lee Co., Ill.

Perhaps no organ or set of organs have as little said about them in medical literature, as those of the alimentary canal from the mouth to the anus.

In the past twelve years I have been called upon to treat diseases of the stomach more than any other part of the human system.

They are developed with various symptoms. One patient says, my stomach aches; again, my stomach is sour or bitter. They complain that their food does not digest, or it will not "lie on the stomach;" or it feels "like a weight in the stomach;" or, the stomach is always "bloated;" "hot wind comes from the stomach;" it "burns in the throat." "I feel something moving in my stomach," says another; "I believe there is something alive in it;" "I have such a beating in my stomach," to use an everyday expression. Says another, "my stomach feels raw, as though there were sores in it." "My stomach is cold," says another. "My stomach is constantly sick," says another, "after my meals; food distresses me so much, I am in constant misery." In this way I might enumerate the various symptoms by the way they express their feelings.

As it runs, two-thirds of the people have some trouble with the digestive apparatus. Some have constantly a bad taste in their mouth; their tongue is dry in the morning; they have a bad breath; the tongue is constantly coated; sometimes it is white, and again brown and red on the sides. I think that two-thirds of the diseases flesh is heir to come first and foremost from some derangement of the digestive apparatus.

*Causes.*—These may come from various sources. Overloading the stomach; giving it too much to do, is one cause. Not having meals at regular intervals; the drinking of too much tea and coffee; eating too fast; not well masticating the food; the use of narcotics, as tobacco; too violent exercise immediately after eating; the drinking of ice-water when one is very warm, giving a too sudden reaction; eating too many sweetmeats; the eating of ice-cream when one is very warm, producing a stasis in the circulation of the vessels of the stomach; the too free use of spirituous and malt liquors, causing congestion, thereby arresting digestion and assimilation.

*Diagnosis.*—We find as much error in diagnosis in these cases as any other; and I might say more. A case or two to illustrate my point: About one year ago, a Mrs. S., who had consulted other physicians before, came to me. She was told by them that she had uterine disease. I got the history of her symptoms. She said that she had constant distress in her stomach; so much so that she could not rest at night. She said that she felt a faint sickness at times; the pain went through to her back. I diagnosed the case gastric dyspepsia. My treatment consisted in the use of anodynes to control the pain; I prescribed chloroform in from five to ten drop doses, to allay pain. To overcome constipation I gave her rhubarb every day for some time. For the gastric trouble I gave her charcoal and bismuth subnitrate, in doses varying from five to ten grains of the former to about the same of the latter, three times a day, before meals, combined in powder. In a short time all of the symptoms disappeared, and she pronounced herself a well woman.

Again, another error in diagnosis: a Mrs. B., whom I was called to see in consultation with another practitioner of about thirty years' experience. The woman seemed to be in a critical condition; she was lying covered with a cold perspiration; extremities cold; pulse about a hundred and forty, and to all appearances seemed to be in a dying condition from some terrible malady.

There seemed to be a tumor pulsating over the region of the stomach. My colleague pronounced it aneurism of the abdominal aorta. I could not agree with his diagnosis. I told him that I would call it gastritis with ulceration of the stomach, but as it was we could not agree. He then said that if I thought that I knew more about the case than he did, I might treat it. I gave aconite, combined with digitalis, to control the circulation. I then prescribed for her large doses of charcoal and subnitrate of bismuth, combined in powder. I gave water of slippery elm to drink, and a light diet. She was the subject of habitual constipation, for which I gave rhei tinct. combined with belladonna. I continued this treatment for some time. She improved: the pulsations ceased, the aneurism, so-called, disappeared. In about three months she was nearly a well woman, none of the symptoms again returning. I might enumerate more cases, but these, I think, are enough to illustrate my point.

